# Pharmacophore Modeling Using Machine Learning for Screening the Blood–Brain Barrier Permeation of Xenobiotics

**DOI:** 10.3390/ijerph192013471

**Published:** 2022-10-18

**Authors:** Saurav Kumar, Deepika Deepika, Vikas Kumar

**Affiliations:** 1 Environmental Engineering Laboratory, Departament d’ Enginyeria Quimica, Universitat Rovira i Virgili, Av. Països Catalans 26, 43007 Tarragona, Spain; 2 Institut d’Investigació Sanitària Pere Virgili (IISPV), Hospital Universitari Sant Joan de Reus, Universitat Rovira I Virgili, 43201 Reus, Spain

**Keywords:** blood–brain barrier, P-glycoprotein, neurotoxicity, graph neural network, machine learning, pharmacophore

## Abstract

Daily exposure to xenobiotics affects human health, especially the nervous system, causing neurodegenerative diseases. The nervous system is protected by tight junctions present at the blood–brain barrier (BBB), but only molecules with desirable physicochemical properties can permeate it. This is why permeation is a decisive step in avoiding unwanted brain toxicity and also in developing neuronal drugs. In silico methods are being implemented as an initial step to reduce animal testing and the time complexity of the in vitro screening process. However, most in silico methods are ligand based, and consider only the physiochemical properties of ligands. However, these ligand-based methods have their own limitations and sometimes fail to predict the BBB permeation of xenobiotics. The objective of this work was to investigate the influence of the pharmacophoric features of protein–ligand interactions on BBB permeation. For these purposes, receptor-based pharmacophore and ligand-based pharmacophore fingerprints were developed using docking and Rdkit, respectively. Then, these fingerprints were trained on classical machine-learning models and compared with classical fingerprints. Among the tested footprints, the ligand-based pharmacophore fingerprint achieved slightly better (77% accuracy) performance compared to the classical fingerprint method. In contrast, receptor-based pharmacophores did not lead to much improvement compared to classical descriptors. The performance can be further improved by considering efflux proteins such as BCRP (breast cancer resistance protein), as well as P-gp (P-glycoprotein). However, the limited data availability for other proteins regarding their pharmacophoric interactions is a bottleneck to its improvement. Nonetheless, the developed models and exploratory analysis provide a path to extend the same framework for environmental chemicals, which, like drugs, are also xenobiotics. This research can help in human health risk assessment by a priori screening for neurotoxicity-causing agents.

## 1. Introduction

The blood–brain barrier (BBB) is a dynamic physiological interface that controls the permeation of xenobiotics from the blood to the central nervous system (CNS) [1,2]. This is essential to protecting the brain from harmful chemicals, viruses, and bacteria, but at the same time, it becomes a hurdle for treating neurodegenerative diseases like schizophrenia, Alzheimer’s, Parkinson’s, etc. Often, drugs developed for targeting CNS fail due to low bioavailability because of the presence of the BBB. The BBB mainly constitutes endothelial cells along with mural cells, immune cells, glial cells, and astrocytes, creating a tight junction that prevents the passive diffusion of xenobiotics [1]. The BBB is also equipped with multiple protein complexes, such as P-glycoprotein (P-gp), choline transporter, breast cancer resistance protein (BCRP), etc. which play a crucial role in active transport [3]. The presence of efflux transporters serves to transfer the compound from the brain, thus acting as a hurdle in CNS drug permeation. P-gp is the most studied efflux transporter, and is responsible for drawing molecules back to blood circulation from CNS; thus, it prevents the therapeutic action of many drugs [1,3]. Structurally, P-gp consists of four domains; two of the hydrophobic transmembrane domains span the lipid bilayer six times to make a channel-like structure that is responsible for substrate binding. The other two hydrophilic nucleotide-binding domains are present on the cytoplasmic face of the membrane, which regulates the ATP metabolism for active transport [3]. Although most lipophilic molecules with smaller sizes (molecular weight < 400 KDa) can cross the BBB in significant amounts by passive diffusion, molecules with a higher molecular weight that are hydrophilic in nature face difficulty in doing the same. As a consequence of this, from the extensive number of chemicals, which is estimated to consist of 10^60^ compounds, only 2% of chemicals are used to make CNS-specific drugs [4].

During the past decades, several rules and in silico models were proposed based on the physicochemical properties of already marketed CNS drugs [4,5,6,7]. To a certain extent, these in silico models help in the selection of ligands for CNS drug development. With recent algorithmic advancements, new methodologies based on graph theory were developed to incorporate the 2D and 3D features of the ligands [7]. Building on this generalized classical ligand descriptors-based modeling, new methods that incorporate target receptors, such as pharmacophore modelling, are widely applied in computer-aided drug design.

Pharmacophore modelling has not been explored much in CNS screening; meanwhile, in bioactive molecule screening, it is used very often [8]. The pharmacophore model is based on the interaction between ligands and receptors. These interactions include all the information related to the structural, spatial, and chemical properties that are responsible for specific pharmacological actions [8,9]. The interaction mostly involves non-covalent bonding, such as hydrogen bonding, pi-pi stacking, the ion–dipole interaction, etc. There are two types of pharmacophore modelling, one a ligand-based type and the other a receptor-based type, depending on the involvement of the ligand and receptor during calculation [8]. Due to the high level of abstraction, pharmacophore features provide some advantages for building robust models [9]. Many commercial software packages are available, such as ligandscout, Ludi, HS-pharm knowledge base, etc., for the pharmacophore calculation and screening of ligands based on the desired receptor. However, none of them are used for CNS-ligand-screening purposes.

In this work, we investigated the pharmacophoric modeling approach for screening the blood–brain barrier permeation of xenobiotics. The ligand-based and receptor-based pharmacophoric features were generated using a custom method. The generated fingerprints were tested on classical machine-learning algorithm models along with a common molecular fingerprint to compare their predictive power. To the best of our knowledge, the pharmacophoric aspect has never been addressed in the case of BBB permeation modelling. The implementation of different models also compares the performance of pharmacophore features with traditional descriptors/fingerprint methods for BBB classification.

## 2. Materials and Methods

### 2.1. Overall Methodological Concept 

**Figure 1:**
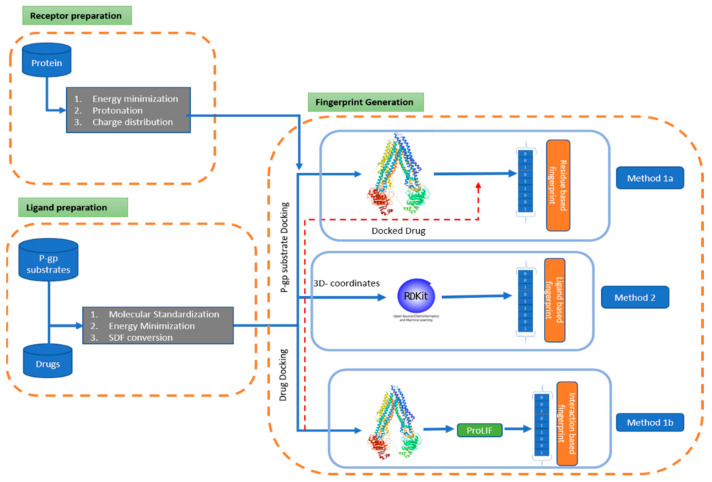
Methodological framework for pharmacophore generation. First, the protein is passed through a pre-processing phase, which includes energy minimization, protonation, and the distribution of charges. Along with this, drugs and P-gp substrates are standardized and finally passed through the energy minimization process to obtain a stable 3D structure. Using these pre-processed data, three types of fingerprints are generated. 1a. Residue-based fingerprint: P-gp substrates are docked and interacting amino acids are extracted; simultaneously, the docked drug interaction residues are mapped over it to generate a fingerprint. Red-dashed arrow represent mapping of docked-drug interaction residues with P-gp interaction residues.1b. Interaction-type fingerprint: drug molecules are docked, and then the docked data are processed with the ProLIF library 2. Ligand-based Fingerprint: drug data, along with 3D coordinates, are processed in Rdkit.

**Figure 2:**
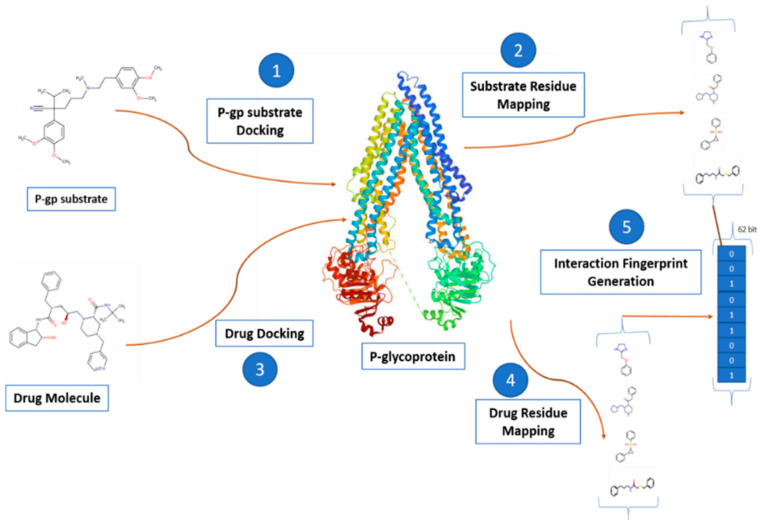
Residue-based pharmacophore fingerprint: DockedFP(1a). (1) The P-gp substrate is docked with the receptor. (2) The active site residue interacting with the P-gp substrates is mapped. (3) The drug is docked with the P-gp receptor. (4) The residues of the active site binding to the drug are mapped. (5) A custom python loop to generate an interaction fingerprint using both the P-gp substrate and drug mappings.

**Figure 3:**
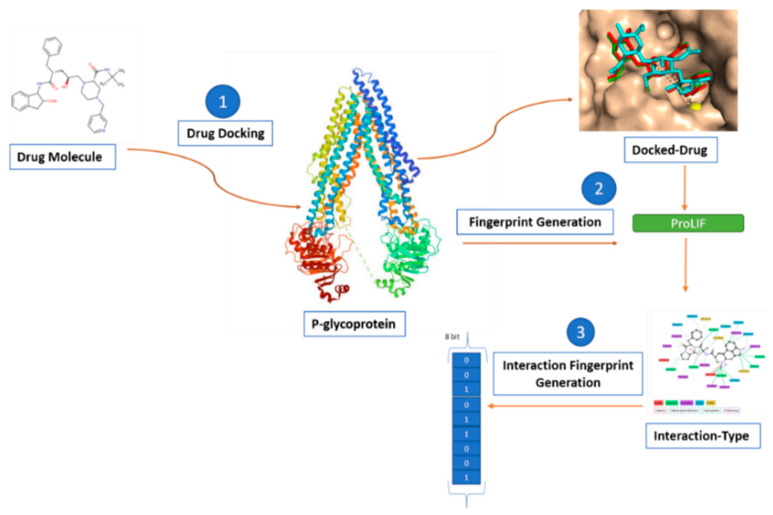
Interaction-type pharmacophore fingerprint: DockedFP(1b). (1) Standardized and stabilized drug molecules are docked with the P-gp receptor. (2) The docked drug data and P-gp receptor are imputed in the ProLIF library. (3) Data are processed using the default distance boundaries given for different interaction types and, finally, the 9-bit fingerprint is generated.

This study aimed to investigate the effectiveness of different fingerprints generated based on pharmacophore modeling in deciphering the blood–brain barrier permeation of xenobiotics. With pharmacophore modeling, we attempted to incorporate the influence of the P-gp protein in the transportation process. To achieve this objective, our study was designed according to the following steps (Figure 1):

- The collection of chemical data from reviewed literature sources, followed by a filtering and standardization process to obtain a stabilized 3D structure.
- A scaffold of the collected data was generated to analyze the distribution of the core structure of the chemical responsible for permeability.
- Stabilization and hydration of the protein retrieved from the protein data bank (PDB) was undertaken, for docking purposes.
- Three methods were implemented to generate pharmacophore fingerprints; among them, two belong to the receptor-based method and one to the ligand-based method.
  (a) The residue-based pharmacophore was generated by docking the P-gp substrates and extracting the most common residues involved in the interaction. The residues were then mapped with the interaction of the drug molecule to generate a 62-bit fingerprint (Figure 2).
  (b) The interaction-type pharmacophore was generated using the docked drug data, which was further processed with the proLIF library to generate a 9-bit fingerprint (Figure 3).
  (c) The 39,971-bit-long ligand-based pharmacophore fingerprint was generated using Rdkit.
- The generated fingerprint and classical fingerprint were then trained on a classical algorithm, such as Support Vector Machine (SVM), RF (Random Forest), and naïve Bayes, for comparison. A newly developed graph model was also implemented for comparison.

### 2.2. Data Collection and Scaffold Generation

For the present work, a wide range of data were reviewed from multiple sources [4,5,6,7]. We found that the recent paper from Fanwang et al. [10] contains all the data from our reviewed sources, so this database was considered for our study. The dataset comprised 7807 molecules in SMILES (Simplified Molecular Input Line Entry System) [11] notation with 4956 molecules as BBB+ and 2851 molecules as BBB-. The dataset is curated from more than 50 peer-reviewed papers, which leads to the heterogeneous endpoint of molecules. Some of the endpoints are encoded as a binary value, with 1 depicting “permeable” and 0 representing “non-permeable”. Meanwhile, in other studies, permeability is represented as a continuous logBB value (concentration in brain/concentration in blood). Various threshold values for logBB were defined to include or exclude molecules in either of the categories. The threshold value is discussed in the respective database paper [10]. To filter duplicate molecules, an extended connectivity fingerprint (ECFP4) [12] of compounds was generated and, using this fingerprint, Tanimoto’s score [13] was calculated. Based on the Tanimoto score, similar molecules were grouped, and a single element from each group was selected upon manual inspection. After the filtering processes, the data size was reduced to 3337 molecules. It was observed that most of the duplicate molecules were stereoisomers, and these stereoisomers were explicitly added to the database, as mentioned by the author. The stereochemistry of the molecules is crucial for defining the various properties of molecules, as well as interactions with the receptors, but in this work, stereoisomers are neglected to reduce excessive computational costs. Filtered molecules were standardized by stripping salt, neutralizing molecules, and converting SMILES into the canonical form using the Molecule Validation and Standardization (MolVS) library [14]. Additionally, single atom molecules, such as Kr, Ne, C, Li, O, Ar, Xe, etc., which do not add anything to the organic drug domain, were removed from the database. Moreover, molecules whose 3D structures could not be generated and optimized in 5000 steps through the steepest descent algorithm using the MMFF94 forcefield [15] were neglected. The optimization of molecules was undertaken using Open Babel’s [16] command-line tools. The final library of 3019 molecules in SDF (structure-data file) format was curated with 1197 molecules as BBB+ and 1822 as BBB-. A filtered and optimized molecular database is publicly available on github. 

In addition, 358 P-gp substrates (encoded in SMILES notation) were curated from Drug Bank [17]. These molecules aid in the generation of amino acid residues maps of the active site (Figure 2). This residue map helps to generate a receptor-based pharmacophore fingerprint (further discussion in Section 2.5.1 method DockedFP 1a). During the pre-processing of P-gp substrates, 57 molecules failed in the 3D geometry optimization. The remaining structures were stored as an SDF library for docking. The P-gp dataset is publicly available on github. 

For scaffold analysis of drug molecules, we used the SMILES of molecules to generate the Murcko scaffold framework [18] of each drug molecule using Rdkit. The Murcko scaffold is a graph-based structure enumeration method; it dissects the cyclic molecule into four units, i.e., rings, framework, linker, and side chains. The generated scaffold was grouped based on its structural similarity. BBB permeability probability for the scaffold was calculated for analysis. For concise and interpretive visualization of high-dimensional drug space, the TMAP [19] algorithm developed by Raymond’s lab was used. The TMAP visualization file and its code are available in the github repository.

### 2.3. P-gp Receptor Preparation 

The protein model of P-gp was retrieved from the Protein Data Bank [20] with ID 6fn1. The structural resolution of the protein was 3.58 Å. The stereochemical validation to investigate the ϕ−ψ dihedral angle in the Ramachandran plot was conducted using a new version of PROCHECK software (European Bioinformatics Institute, Cambridge, UK) [21]. The Ramachandran plot statistics showed 88.1% (1270) and 11.6% (167) residues in the most favored regions and allowed regions, respectively (Figure S1). Meanwhile, the expected value for comparison of the structure with 2 Å of resolution was more than 90% in the most favored region. Docking preparation was initiated by energy minimization of the protein using ModRefiner [22], which helps to achieve the lowest energy conformation of a molecule. The energy minimized structure has a TM (template modeling) score of 0.9946, as compared to the initial structure, which shows that there is almost no loss in fold during energy minimization. A python script “prepare_receptor.py” from AutoDock Tools [23] was used to protonate and redistribute Kollman partial charges over the stabilized protein. Finally, the protein was converted to the ready-to-dock format of AutoDock Vina [24], i.e., pdbqt (Protein Data Bank, partial charge, and atom type). It is well known that P-gp has multiple binding sites, such as the H-site (binding Hoechst 33,482), the R-site (binding rhodamine 123), and the P-site (binding prazosin and progesterone), etc. [25]; however, until now, there was no database of P-gp substrates with the specific binding site available. Hence, we considered the previous work on structural and functional aspects of P-gp, which mentioned that the transmembrane helices from 4–6 and 10–12 are mainly involved in substrate binding [26,27]. As such, the center coordinates (X = 160 Å, Y = 145.4 Å, Z = 142.88 Å) with a grid box size of 40 Å between these transmembrane helices were considered. 

### 2.4. Ligand Preparation

The optimized ligands SDF library was processed using a python script “mk_prepare_ligand.py” from the Meeko library [28]. Meeko is a python-based library for the preparation of small molecules for docking, developed by the Forli lab at the Center of Computational Structural Biology (CCSB) at Scripps Research. This script carries out all the necessary pre-processing, such as the distribution of charges (Gasteiger charges), the addition of polar hydrogen atoms, and, finally, the conversion from the PDB format to the PDBQT format. 

Once the raw material required for docking was assembled, two types of pharmacophore fingerprints were developed, i.e., ligand-based and receptor-based fingerprints (residue-based and interaction-type-based fingerprints). For the ligand-based type, the pre-developed method from Rdkit was implemented, while AutoDock Vina and Python were used to generate the custom structure-based interaction fingerprint.

### 2.5. Interaction Fingerprint Generation 

#### 2.5.1. Receptor-Based Fingerprint

##### Method DockedFP(1a): Residue-Based Type

High throughput docking of the Drug Bank’s P-gp substrate to the binding site of P-gp was achieved using python (ver.3.7, Python Software Foundation, Wilmington, Delaware, USA) [29]; under the hood, it implements the Autodock-vina command in a parallel fashion (Figure 2). While docking, 31 molecules were neglected due to an error in atom-type notation. Properly docked ligands were then processed through the “process_VinaResult.py” script of the AutoDock tools repository to extract the list of residues responsible for binding with the drug molecule. Then, the 62 most common residues of active binding sites were extracted. A plot of the most common residues is presented in the Supplementary File (Figure S2). For mapping, a python dictionary of these 62 residues was built. The optimized SDF library of the drugs was then processed through the ligand preparation stage and docked at the narrow channel of the P-gp receptor. Interacting residues were then extracted and looped through the mapping dictionary to generate a 62-bit vector array, where 1 and 0 represent the presence and absence of interaction.

##### Method DockedFP(1b): Interaction-Type-Based Fingerprint

Interaction-type fingerprint generation implements the 9-bit array containing only bonding-type information, such as cationic, anionic, hydrophobic, hydrogen, etc. The ProLIF library [30] was used to encode interaction-type information as a fingerprint (Figure 3). ProLIF is a python-based library developed by the Chemosim-lab; it is used to generate interaction fingerprints for complexes including ligands, proteins, DNA, or RNA, based on the data extracted from molecular dynamics, docking simulations, and experimental structures. PDBQT files of drugs and p-gp docking were processed through the “mk_copy_coords.py” script of the meeko library for SDF conversion. SDF files, along with receptor proteins, were then fed with the default threshold distance for each interaction. Finally, the library generated the interaction-type array as a bit vector with 0 and 1 depicting absence and presence.

#### 2.5.2. Ligand-Based Generation

##### Method 2: Rdkit Pharmacophore Fingerprint

For the ligand-based pharmacophore generation, the structure of the receptor was not taken into consideration [9]. It solely relied on the features of ligands that contribute to the interaction with the receptors. Ligand-based pharmacophore generated by Rdkit leads to 39,971 long-bit array fingerprints. The generated fingerprints consist of features such as hydrophobicity, donor capacity, affinity, acidic group, basic group, aromatic attachment, etc. As compared to ECFP4 fingerprint generation, this fingerprint is much more memory intensive, but it includes all the information of a molecule, just like the ECFP4 fingerprint. The size of the fingerprint can be reduced using PCA (principle component analysis) and newly developed deep learning methods such as Auto-Encoders, which take high-dimensional feature size as the input and returns low-dimensional representations while preserving the information and semantics. 

### 2.6. GNN Implementation

**Figure 4:**
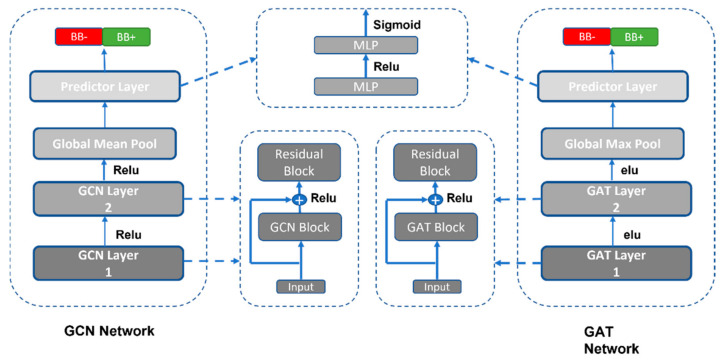
The architecture of the graph neural network models. The GCN and GAT networks share almost the same architecture; the main difference is in their block layer, i.e., the GCN block performs the convolution to learn the low-level representation of the molecular graph, while GAT implements multi-headed attention with convolution to learn the weighted representation of the molecular graph. The GCN network uses a Relu (rectified linear activation unit) as an activation function while GAT implements elu (exponential linear unit) between the layer stacking. Finally, for the downstream task, both implement the same architecture of the predictor layer. The dashed line represents the internal architecture of each block in the network.

Two variants of graph neural networks (GNN), that is, the graph convolution network (GCN) [31] and the graph attention network (GAT) [32], were trained for 200 epochs at a batch size of 256 with the Adam optimizer at a learning rate of 0.001. The optimal configuration (Figure 4) of GCN consists of two feature extractor layers embedded with a residual block, followed by a non-linear transformation block in each. The extractor layer was followed by a non-linearly transformed predictor layer with a sigmoid as an activation layer for output. While the GAT network is a combination of two multi-headed Graph attention layers as feature extractors with elu (exponential linear unit) activation, the predictor layer is the same as GCN. The hyperparameters of both networks are tabulated in Supplementary Table S1. To build neural networks, blocks of graph operation layers from the PyTorch geometric library were compiled into the whole graph neural network architecture using PyTorch [33]. The DeepChem library [34] was used to populate the node features vector of the molecular graph. The fundamental features of the molecules were atom type, formal charge, hybridization, hydrogen bonding, aromaticity, degree/connectivity, number of hydrogens, and partial charge; these features were encoded in a one-hot feature vector to make it easy for algorithms to find the global minima during learning.

### 2.7. Model Validation

After ensuring a clean and representative compilation of the dataset, three algorithms were trained using different representations of pharmacophore interactions and molecular features, alone or in combination. For training and experimental measurement across a variety of drug discovery projects, machine learning models, including the support vector machine (SVM) [35], a tree-based model such as random forest (RF) [36], and Bayesian statistics, were used . To ensure the robustness of the predictive results, all classical machine learning models were trained and evaluated through an exhaustive 5-fold stratified cross-validation method. This means the whole dataset was divided into 5 subsets with equally distributed classes, and 4 sets among them were used for training, while the remaining one was for validation. The classical algorithms were implemented using the popular python scikit-learn [37] machine learning package. A comparison of the performance of these models with newly emerged GNN variants was conducted. 

### 2.8. Evaluation Metrics

To evaluate the results of the model, several common classification metrics, as mentioned in Equation (1)–(5), where *TP* refers to true positive, *TN* to true negative, *FP* to false positive, *FN* to false negative, and *MCC* to Matthews correlation coefficient.

(1) $$Accuracy=\frac{TP\;+\;\;\;TN}{Total\;samples}$$

(2) $$Precision=\frac{Number\;of\;TP}{TP+FP}$$

(3) $$\;\;Recall=\frac{Number\;of\;TP}{TP\;+\;FN}$$

(4) $$F1\;score=2*\frac{Precision\;*\;Recall}{Precision+Recall}$$

(5) $$MCC=\frac{TP\times TN-FP\times FN}{\sqrt{\left(\left(TP+FP\right)\left(TP+FN\right)\left(TN+FP\right)\left(TN+FN\right)\right)}}$$

The AUC-ROC score were calculated using the scikit-learn package in python. The most common metric used for model evaluation is accuracy (Equation (1)), but this statistic is not suitable for unbalanced datasets. Precision measures what proportion of BBB+ classes are actually positive (Equation (2)). Other metrics, such as recall/sensitivity (Equation (3)) assess the model’s ability to predict actual BBB+ molecules as positive. It is mainly dedicated to assessing false-negative classes. The harmonic mean of precision and recall gives an F1 score that helps in comparing different classifiers (Equation (4)). The Matthews correlation coefficient (MCC) is used to measure the quality of binary classification and multiclass classification (Equation (5)). It also handles unbalanced classes. The Area Under the Receiver Operating Characteristics (AUROC) score measures the performance of the classification model. ROC represents the probability curve, while AUC represents the degree and measure of separability [38].

The processing of molecules for optimization, validation, and docking was undertaken using a Dell server with two 8 cores of Xenon processors running at 3.29 GHz, with 128 GB of RAM. For classical and graph-based machine learning 16 GB, NVIDIA RTX A4000 GPU was also leveraged.

## 3. Results

### 3.1. Scaffold-Based Chemical Space Analysis

**Figure 5:**
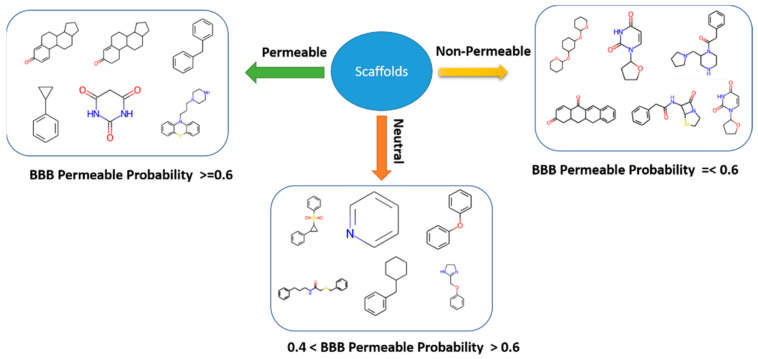
Grouping scaffold based on BBB permeability probability. Scaffolds with permeability probability above 0.6 and below 0.6 are assigned as permeable and non-permeable scaffolds, respectively. Scaffolds between 0.4 and 0.6 are classified as neutral.

For the first time, structural diversity analysis of the drugs was conducted to determine a priori whether a compound’s backbone/scaffold is suitable for crossing the barrier or not. During scaffold analysis, we found that, in the chemical space of 3337 molecules, for 196 molecules, no scaffold was generated as they were acyclic, while the remaining 1864 were cyclic molecules. In total, 79.2% of the scaffold clusters generated from cyclic compounds consist of only one unique molecule. Meanwhile, in the remaining scaffolds, molecule distribution ranges from 2–200 molecules in the scaffold group, as shown in Figure S3. As expected, benzene was found to be the most common scaffold, which suggests that molecules possessing rigid structures can be used as a good base for optimizing lead candidates [39]. Furthermore, to assess the permeability power of each scaffold, the permeable probability of each scaffold was calculated. Scaffolds with a probability above 0.6 were assigned as permeable scaffolds, while those below 0.4 were designated as non-permeable (Figure 5). Scaffolds with a probability between 0.4 and 0.6 were considered neutral. The probability score of some top scaffolds is noted in Table S2. This categorization provided a broad picture of classifying a molecule’s permeability on the basis of its scaffold, which can be helpful in narrowing down the ligand space. In addition, a molecular framework with a high probability may be considered as a starting point for de novo BBB permeable drug development, whose therapeutic nature can be further tuned by adding favorable substituents by a medicinal chemist. Other parameters, such as polar surface area, molecular weight, lipophilicity, flexibility, and hydrogen bond donors, were consistently identified as crucial properties for tweaking BBB permeability for drug design [39]. We found that addition or deletion in functional groups over the scaffold base may lead to changes in the above-mentioned parameters, which eventually leads to an increase or decrease in permeability. This phenomenon was visualized using TMAP, showing the arrangement of each drug molecule around its common denominator scaffolds and the change in the permeability with slight modification in substituents. For example, the incorporation of substituent fluorine in scaffolds enhanced the lipophilicity of molecules and reduced the efflux ratio [39]. Some most common permeable scaffolds, excluding benzene, have structures/substructures very similar to Gona-1,3,5(10)-trien-3-ol, Diphenylmethane, Benzodiazepine, Cyclopropyl benzene,2,4,6-Pyrimidinetrione, etc., possessing rigid systems (Figure S2). It may be possible that these common permeable scaffolds have low TPSA, suggesting that TPSA can be used as a parameter for differentiating between CNS and non-CNS drugs [39,40]. However, in our analysis, we did not find major differences in TPSA distribution between permeable and non-permeable, which requires further investigation to reach a conclusion (Figures S4–S6). Apart from TPSA, other defining properties, such as the hydrogen bond donor (HBD) and the hydrogen bond acceptor (HBA), may also attribute to the permeability (Figures S4–S6), but it depends on the functional group substituted over the scaffold [39]. In this work, we did not choose multiple parameters for analysis, but there is scope to investigate it further. In short, scaffold analysis can be considered the first step in the virtual screening of drugs.

### 3.2. Classical ML Models

Four different algorithms based on classical learning were implemented to build predictive models using chemical fingerprints, pharmacophoric features, or a combination of both (Table 1). Receptor-based generated pharmacophore fingerprints were first tested on a dummy model, which randomly selects class labels from the uniform distribution of datapoints. The dummy model gives 50% accuracy for methods DockedFP (1a)- and DockedFP (1b)-generated fingerprints. This was considered as a baseline to validate whether the generated fingerprints hold any relevant information or not. Other models trained using DockedFP (1a) and DockedFP (1b) performed better than the baseline model with 60–63% accuracy and a 69–74% F1 score, but lower in comparison to the ECFP4 fingerprint, which showed 72–76% accuracy and a 78–81% F1 score. This might be because the size of the ECFP4 fingerprint is 1024 bits long, hence it contains more elaborate information about topological properties, substructure information, and common molecular features such as stereochemical information, which is essential to define the biological endpoint of the molecule, as is the case for QSAR models [4,5,6]. In contrast, the pharmacophore fingerprints of the DockedFP (1a) method and the DockedFP (1b) method have 62- and 9-bit vector sizes, which contain limited information only about the binding site residues and the type of interaction, but it is essential information in case of pharmacophore data. 

Furthermore, the ECFP4 fingerprint was merged with both structure-based generated fingerprints to mitigate this lack of information. In combination, the performance of the combinational fingerprint is similar (72–76% accuracy) to the ECFP4 fingerprint, which shows that the addition of pharmacophoric features to the structural fingerprint does not enhance its predictive power. This could be due to the dominance of ligand-based molecular fingerprints over pharmacophoric features developed by custom methods. This is because the algorithms trained solely on structural pharmacophoric have significant predictive power (61–63%), but, when merged with higher-order-sized fingerprints, it is considered to be noise and is therefore neglected by the algorithm. We also tried adding continuous descriptors (molecular weight, partition coefficient (Log P), topological polar surface area) with pharmacophoric features, but this resulted in a decline in model performance (result not shown). Because of their continuous values and the combination of the continuous and binary values, the addition of descriptors might cause this decline in performance because it confuses the algorithms when learning the relevant pattern, and may therefore reduce the performance of the model. That is why the addition of the ECFP4 fingerprint is preferred over descriptors. The combined model (ECFP4+ DockedFP (1a & 1b)) gave 76% accuracy for the SVM and RF algorithm, while the performance dropped to 72% on Naïve Byes.

The ligand-based pharmacophore generated using Rdkit performs slightly better than the ECFP4 fingerprint with the Random Forest algorithms (77% accuracy) because the ligand-based pharmacophore facilitates the understanding of structural and activity relationships with the target receptor. The ligand-based pharmacophore also achieves the highest precision (78% on RF) among the examined pharmacophores, showing a decrease in false prediction. This satisfies our hypothesis that some molecules have the physiochemical property in the desirable range for permeation but are still not able to permeate because of the involvement of P-gp. By adding ligand interaction to the fingerprint, the model manages to capture this information up to a certain point. There is a decrease in ligand-based pharmacophore performance with the SVM (75%) and Naïve Byes (71%) algorithms. Among the three algorithms, the superior performance of the Random forest is attributed to its tree-like distribution of feature points and the implicit “ensemble learning” which enables multiple decision tree models to train on the same data [36]. Additionally, binary fingerprints complement the features of the random forest as they make it easier for the algorithm to split nodes based on “yes” and “no” until a leaf node is reached. Random forest algorithms are also highly prone to overfitting, so the mindful tuning of hyperparameters is required for their superior performance.

The performance of the structure-based generated pharmacophore is lower than the ligand-based pharmacophore for BBB permeation modelling. This may be explained by the following reasons:

(i) The BBB has multiple efflux transporters, but we considered only P-gp due to limited data availability [1,2].

(ii) For pharmacophore generation, a specific binding site on a protein is crucial, but P-gp has a broad substrate binding site. In this case, a common site is assumed for the binding of all drug molecules [3].

These assumptions were made to simplify the model, which might be the reason for the decline in the performance of the pharmacophore model. Otherwise, pharmacophore-based modeling is at the heart of computer-aided drug design (CADD) modeling [8,9]. It helps to prioritize the promising derivatives from a wide chemical space. Pharmacophore models are often used in virtual screening because they cover different important aspects that are important for the activity of the compound. Commercial tools for pharmacophore-based modeling account for the presence of important physicochemical properties for biological activity and molecular docking to calculate the fitting of the compound [9]. Having both fitting and physicochemical data, the success rate of virtual screening exceeds that of alternative techniques. Pharmacophore modeling for BBB permeation is a challenging task as the permeation process is controlled by multiple parameters and interactions; modeling all the interactions simultaneously is challenging due to limited data availability.

### 3.3. Comparison with GNN Models

The application of deep learning in the field of chemicals/drug discovery is highly prevalent in the form of GNN because of the resemblance of the molecular structure to a graph [41]. We found that both convolution and attention networks provided similar results for accuracy (74%), precision (77%), and the F1 score (79%), whereas recall value was higher with convolution (80%) than with attention (78%) (Table 1). Additional metrics, such as the Matthews correlation coefficient (MCC) and the AUC-ROC score, gave better result for convolution with attention (51% of MCC and 75% of AUC-ROC) than alone with convolution (47% of MCC and 72% AUC-ROC). In comparison to classical models trained on pharmacophore features expect ligand-based pharmacophores, GNN variants performs better. Whereas slight decrease in performance against ECFP4 fingerprint. Training of the graph network has been monitored by overfitting sensitive metric “loss value” to choose the training epoch and learning rate. Tweaking the input features of the graph model, such as the addition of interaction-type information from pharmacophore data, might help to elevate its performance. The new variant of GCN-named relational graph convolution networks (RGCN) appears promising for future work with multiple pharmacophores [42]. It can be explored further in the continuation of this work. If GNN leads to improvements in prediction, it can help in screening new biological entities for CNS targeting and exclude molecules that are toxic to the CNS.

## 4. Conclusions

This study investigated whether the addition of the pharmacophoric interaction in the screening process for CNS-targeted screening can elevate the robustness of the predictive models. To perform this analysis, chemical data were curated from different sources and thoroughly filtered to remove redundant molecules. Additionally, the data were passed through a computationally expensive process to generate an optimized 3D structures library of the molecules and P-gp substrates. This SDF molecules library is publicly available for future exploration. The generated data were processed through various methods to create interaction fingerprints. The generated interaction fingerprints and combinations of docked and ECFP4 fingerprints were trained on classical machine learning algorithms. It is concluded that the ligand-based pharmacophore performs better than the traditional ECFP4 fingerprint in classifying permeable and non-permeable groups. In contrast, receptor-based pharmacophore modeling is still not advanced enough for the CNS screening process. We used the custom method to generate pharmacophore fingerprints, while using the commercial tool might help to elevate the predictive performance. This work lays a foundation for pharmacophore modeling using machine learning for CNS screening. Its performance can be further improved in future work by the application of the relational graph convolutional network, a kind of modeling capable of dealing simultaneously with multiple pharmacophores generated from interactions with different proteins and also the physicochemical properties of drugs.

We tried to move one step ahead from the traditional descriptor-based screening method, using protein-drug interactions to evaluate BBB permeability. Until now, in silico models are focused on passive diffusion as a mechanism of drug permeation across the BBB, which may not be true. We tried to incorporate the protein–ligand interaction using a single efflux transporter, but multiple transporters can contribute to the permeation mechanism [1]. In the future, multi-pharmacophore modeling can be implemented to improve predictive capabilities. Currently, the data available regarding active transport through BBB is limited, which restricts our potential to develop a robust in silico model. Even though P-gp is the most studied protein in the domain of efflux transporters, the database of the P-gp substrate with its specific binding site is still not available. Recent advancements in technology and high throughput screening techniques for in vitro and in vivo models will populate data that can be used in the future to make an efficient and reliable model. Nonetheless, this model lays the groundwork for considering active transport along with passive diffusion for BBB permeation. The proposed framework can also support the 3Rs principle, and the use of such methods within read-across approaches [43] or in a wider integrative translational framework for chemical-induced neurotoxicity [44]. The current modeling pipeline is a generic framework that can be applied in the field of human risk assessment, as in silico methods for screening neurotoxic environmental chemicals have not yet been implemented. Screened neurotoxic chemicals space can then be ranked according to their toxic potency by studying their kinetic properties and active concentration in CNS using dynamic modelling methods such as PBPK (physiology-based pharmacokinetics) [44,45]. This integrative approach of QSAR (quantitative structure activity relationship) and PBPK will ease the load of in vitro experiments and will also be helpful in revealing the adverse effects of these toxicants on different organs/system such as the liver, gut, and immune system in a mechanistic way [2,46].

## Figures and Tables

**Table 1:** Comparison of models for the different fingerprints.

Models	Features	Accuracy (Train/Test)	Precision (Train/Test)	Recall (Train/Test)	F1 Score (Train/Test)
Baseline	DockedFP (1a) DockedFP (1b)	0.50/0.50 0.50/0.50	0.60/0.61 0.61/0.61	0.48/0.49 0.49/0.49	0.53/0.54 0.55/0.55
SVM	ECFP4 fingerprint DockedFP (1a) DockedFP (1b) Rdkit Pharmacoprint ECFP4+ DockedFP (1a) ECFP4+ DockedFP (1b)	0.92/0.76 0.62/0.61 0.71/0.63 0.88/0.75 0.92/0.76 0.93/0.76	0.91/0.77 0.62/0.62 0.70/0.64 0.84/0.75 0.91/0.77 0.92/0.77	0.96/0.86 0.93/0.92 0.93/0.87 0.98/0.89 0.96/0.87 0.97/0.86	0.94/0.82 0.75/0.74 0.80/0.74 0.90/0.81 0.93/0.82 0.94/0.81
Random Forest *	ECFP4 fingerprint DockedFP (1a) DockedFP (1b) **Rdkit Pharmacoprint** ECFP4 + DockedFP (1a) ECFP4 + DockedFP (1b)	1/0.76 0.62/0.61 0.91/0.60 **0.99/0.77** 1/0.76 1/0.76	1/0.76 0.62/62 0.90/0.64 **0.99/0.78** 1/0.76 1/0.76	1/0.86 0.92/0.91 0.95/0.73 **0.99/0.84** 1/0.87 1/0.87	1/0.81 0.75/0.74 0.92/0.69 **0.99/0.81** 1/0.81 1/0.81
Naïve Byes	ECFP4 fingerprint DockedFP (1a) DockedFP (1b) Rdkit Pharmacoprint ECFP4 + DockedFP (1a) ECFP4 + DockedFP (1b)	0.76/0.72 0.62/0.62 0.61/0.60 0.72/0.71 0.76/72 0.76/72	0.78/0.75 0.62/0.62 0.66/0.64 0.72/0.71 0.78/0.75 0.78/0.75	0.83/0.80 0.9/0.9 0.75/0.74 0.90/0.89 0.84/0.8 0.84/0.81	0.81/0.78 0.74/0.74 0.70/0.69 0.80/0.79 0.81/0.77 0.81/0.78
Graph Convolution Network (GCN)	Descriptors	0.81/0.74	0.83/0.77	0.85/0.80	0.84/0.79
Graph Attention Network (GAT)	Descriptors	0.83/0.74	0.87/0.77	0.84/0.78	0.85/79

Note: Additional metrics, such as the Matthews correlation coefficient (MCC) and the AUC-ROC score, are tabulated in Supplementary Table S3. * Best performing model is in bold.

## Data Availability

The data and the codes are freely available at https://github.com/Crispae/Pharmacophore-modeling-using-machine-learning-for-screening-Blood-Brain-Barrier-permeation-of-xenobi (accessed on 7 August 2022).

## Supplementary Materials

The following supporting information can be downloaded at: https://www.mdpi.com/article/10.3390/ijerph192013471/s1, Figure S1: Ramachandran plot analysis of P-gp protein (6fn1) using PROCHECK. Figure S2: Frequency plot of most common residues of P-gp active site. Figure S3: Scaffold distribution of the chemical space. Figure S4: Trends in physiochemical properties of BBB permeable and non-permeable molecules permeable scaffold. Density of molecules having properties in certain range is shown along y-axis and value of properties on x-axis. The Bar is colored based on permeable (orange) and non-permeable (purple). Molecular properties plotted here are (i) Molecular Weight (ii) LogP (iii) H bond donors (iv) H bond acceptors (v) logD (vi) TPSA. Figure S5: Trends in physiochemical properties of BBB permeable and non-permeable molecules in non-permeable scaffold. Density of molecules having properties in certain range is shown along y-axis and value of properties on x-axis. The Bar is colored based on permeable (orange) and non-permeable (purple). Molecular properties plotted here are (i) Molecular Weight (ii) LogP (iii) H bond donors (iv) H bond acceptors (v) logD (vi) TPSA. Figure S6: Trends in physiochemical properties of BBB permeable and non-permeable molecules in neutral scaffold. Density of molecules having properties in certain range is shown along y-axis and value of properties on x-axis. The Bar is colored based on permeable (orange) and non-permeable (purple). Molecular properties plotted here are (i) Molecular Weight (ii) LogP (iii) H bond donors (iv) H bond acceptors (v) logD (vi) TPSA. Table S1: Models Hyperparameters. Table S2: Top 4 scaffolds of neuroactive chemical space. Table S3: Models performance on MCC and AUC-ROC metrics.

## Abbreviations

BBB	Blood–brain barrier
BCRP	Breast cancer resistance protein
P-gp	P-glycoprotein
CNS	Central nervous system
SMILES	Simplified Molecular Input Line Entry System
ECFP4	Extended Connectivity Fingerprint
MMFF94	Merck Molecular Force Field
SDF	Structure-Data File
TMAP	Tree MAP
PDB	Protein Data Bank
PDBQT	Protein Data Bank, Partial Charge and Atom Type
GNN	Graph Neural Network
GCN	Graph Convolution Network
GAT	Graph Attention Network
ELU	Exponential Linear Unit
SVM	Support Vector Machine
RF	Random Forest
TPSA	Topological Polar Surface Area
HBA	Hydrogen Bond Acceptor
HBD	Hydrogen Bond Donor
RGCN	Relational Graph Convolution Network
QSAR	Quantitative structure activity relationship
PBPK	Physiologically Based pharmacokinetics
