# Supplementary material for: Pharmacophore Modeling Using Machine Learning for Screening the Blood–Brain Barrier Permeation of Xenobiotics
Source: Int J Environ Res Public Health. 2022 Oct 18;19(20):13471. doi: 10.3390/ijerph192013471 (PMC9602466; doi:10.3390/ijerph192013471)
Supplement: Supplementary file 1 [file ijerph-19-13471-s001.zip › ijerph-1908937-supplementary.pdf]

Pharmacophore modeling using machine learning for screening Blood-Brain Barrier permeation of xenobiotics

Saurav Kumar [a], Deepika Deepika [a] and Vikas Kumar [a,b*]

[a]Environmental Engineering Laboratory, Departament d' Enginyeria Quimica, Universitat Rovira i Virgili, Av. Països Catalans 26, 43007 Tarragona, Catalonia, Spain

[b]IISPV, Hospital Universitari Sant Joan de Reus, Universitat Rovira I Virgili, Reus, Spain

* **Correspondence: Corresponding author: Environmental Engineering Laboratory, Departament d'Enginyeria Química, Universitat Rovira i Virgili, Tarragona, Catalonia, Spain. Tel.: +34977558576.** E-mail address: vikas.kumar@urv.cat

Supplementary

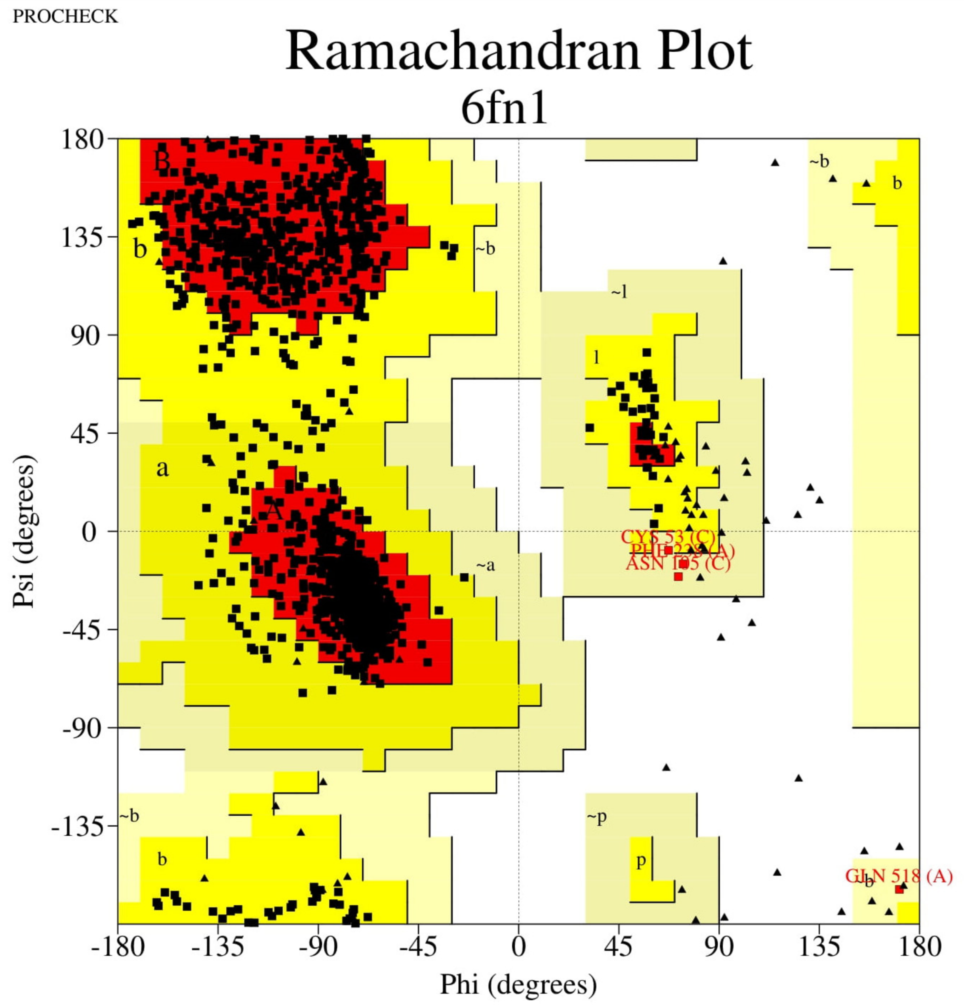

Plot statistics

Residues in most favoured regions [A,B,L]	1270	88.1%
Residues in additional allowed regions [a,b,l,p]	167	11.6%
Residues in generously allowed regions [~a,~b,~l,~p]	4	0.3%
Residues in disallowed regions	0	0.0%
	----	------
Number of non-glycine and non-proline residues	1441	100.0%
Number of end-residues (excl. Gly and Pro)	5	
Number of glycine residues (shown as triangles)	128	
Number of proline residues	53	

Total number of residues	1627	

Based on an analysis of 118 structures of resolution of at least 2.0 Angstroms
and R-factor no greater than 20%, a good quality model would be expected
to have over 90% in the most favoured regions.

6fn1_01.ps

Figure S1: Ramachandran plot analysis of P-gp protein (6fn1) using PROCHECK

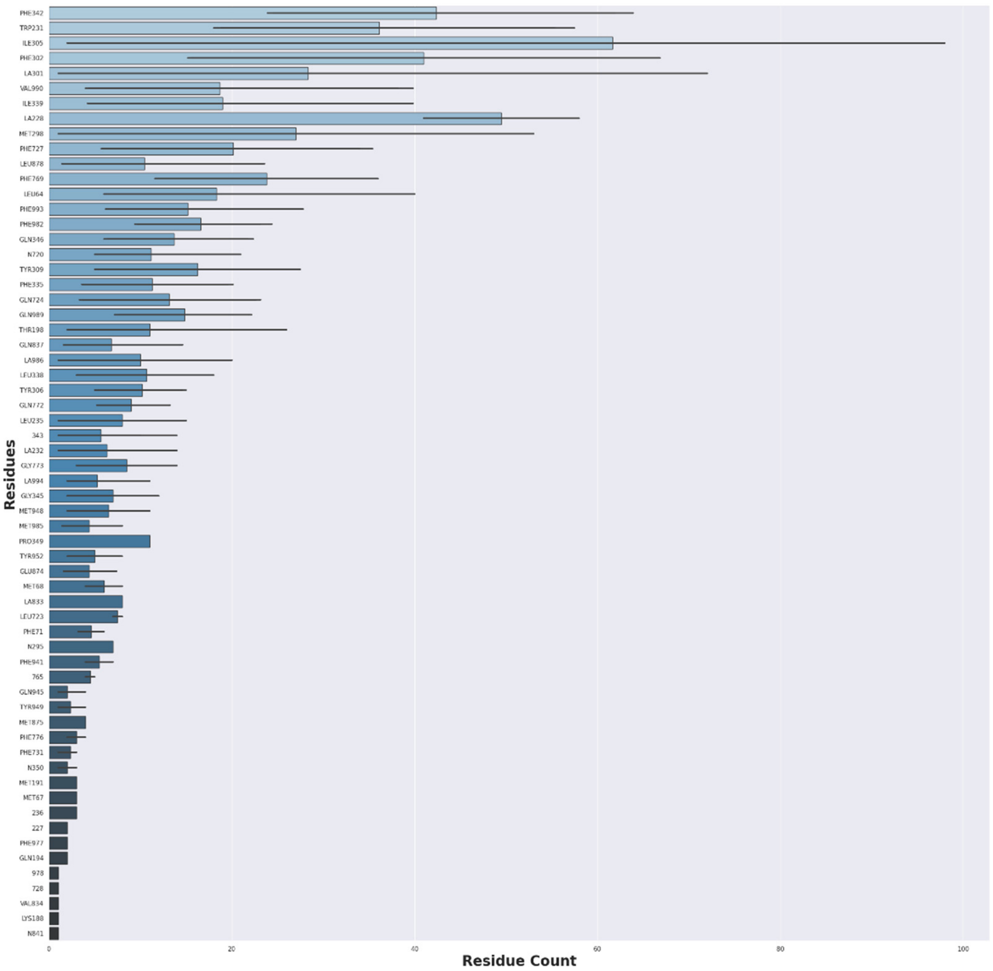

Figure S2: Frequency plot of most common residues of P-gp active site.

Table S1: Models Hyperparameters

Models	Hyperparameters
SVM	Kernel: RBF, C:1
Random Forest	n_estimators: 100, criterion: gini, min_samples_split: 2, min_samples_leaf:1, max_features: sqrt,
Naïve Byes	Gaussian
Graph Convolution Network	Epochs:200, Adam optimizer, learning rate:0.001, criterion: Binary cross entropy, number of layers:2 dropout: 0.6
Graph Attention Network	Epochs:200, Adam optimizer, learning rate:0.001, criterion: Binary cross entropy, Input head:8, output head: 1, concat: False

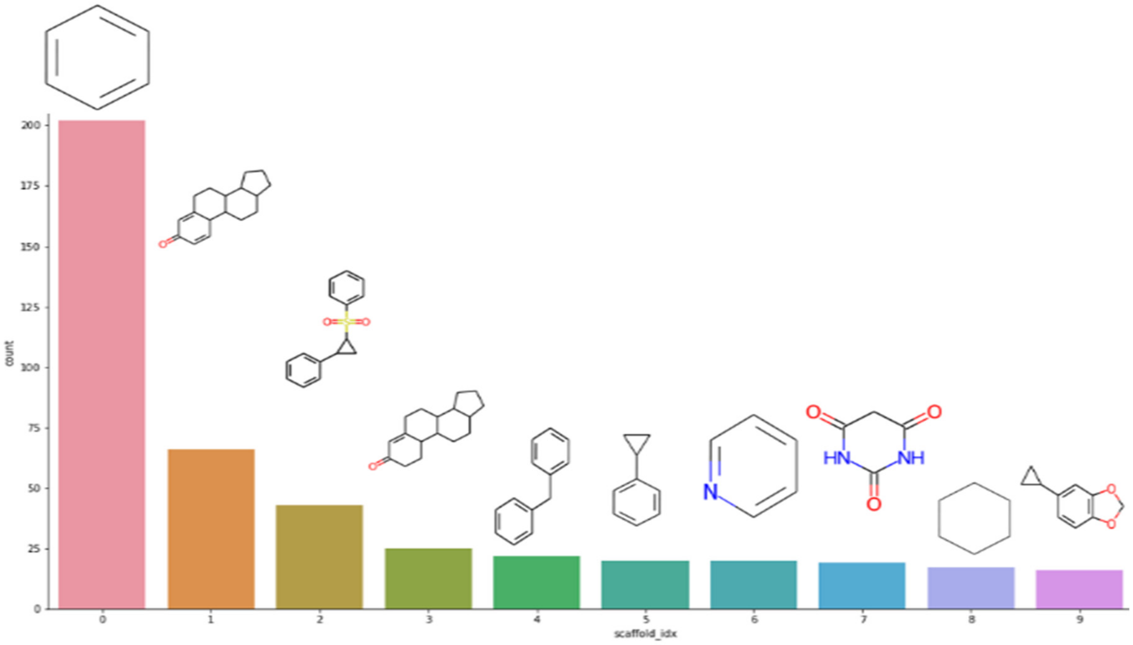

Figure S3: Scaffold distribution of the chemical space

Table S2: Top 4 scaffolds of neuroactive chemical space

Scaffolds	BBB+ count	BBB- count	BBB permeable probability
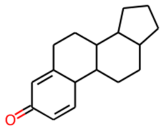	65	1	8.00
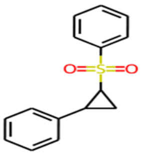	22	21	3.35
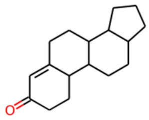	18	4	3.83
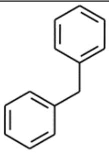	13	7	2.9

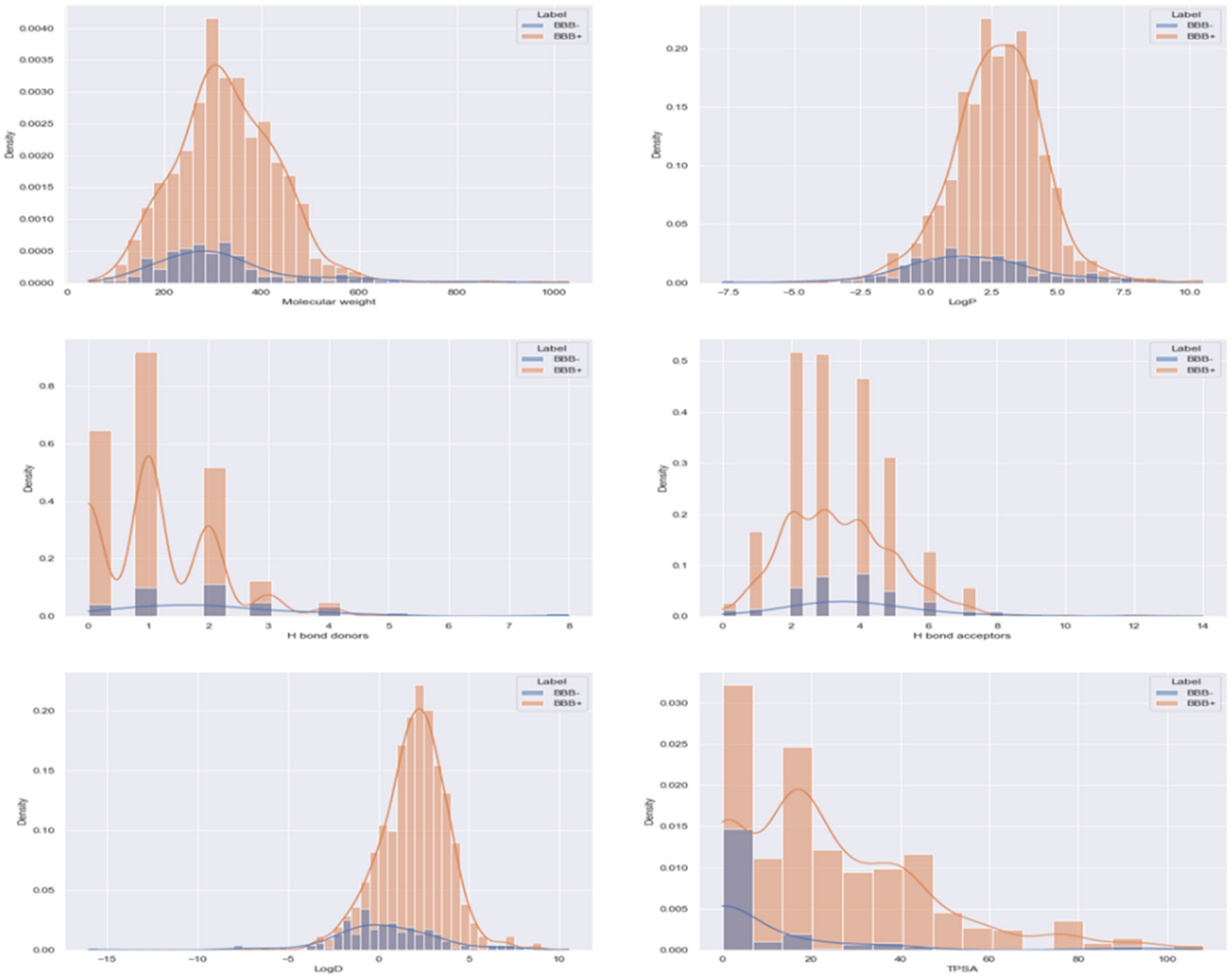

Figure S4: Trends in physiochemical properties of BBB permeable and non-permeable molecules permeable scaffold. Density of molecules having properties in certain range is shown along y-axis and value of properties on x-axis. The Bar is colored based on permeable (orange) and non-permeable (purple). Molecular properties plotted here are i) Molecular Weight ii) LogP iii) H bond donors iv) H bond acceptors v) logD vi) TPSA.

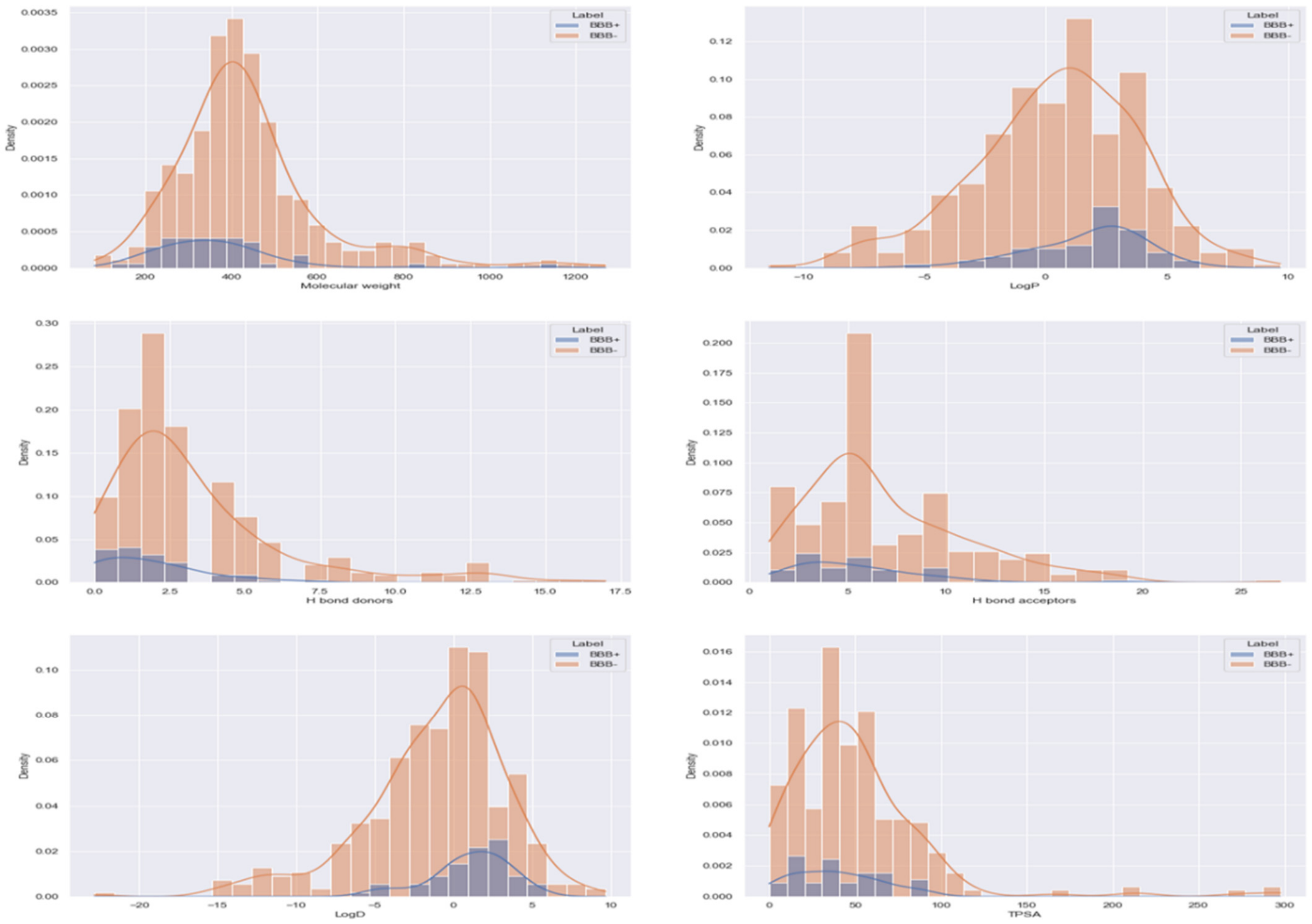

Figure S5: Trends in physiochemical properties of BBB permeable and non-permeable molecules in non-permeable scaffold. Density of molecules having properties in certain range is shown along y-axis and value of properties on x-axis. The Bar is colored based on permeable (orange) and non-permeable (purple). Molecular properties plotted here are i) Molecular Weight ii) LogP iii) H bond donors iv) H bond acceptors v) logD vi) TPSA.

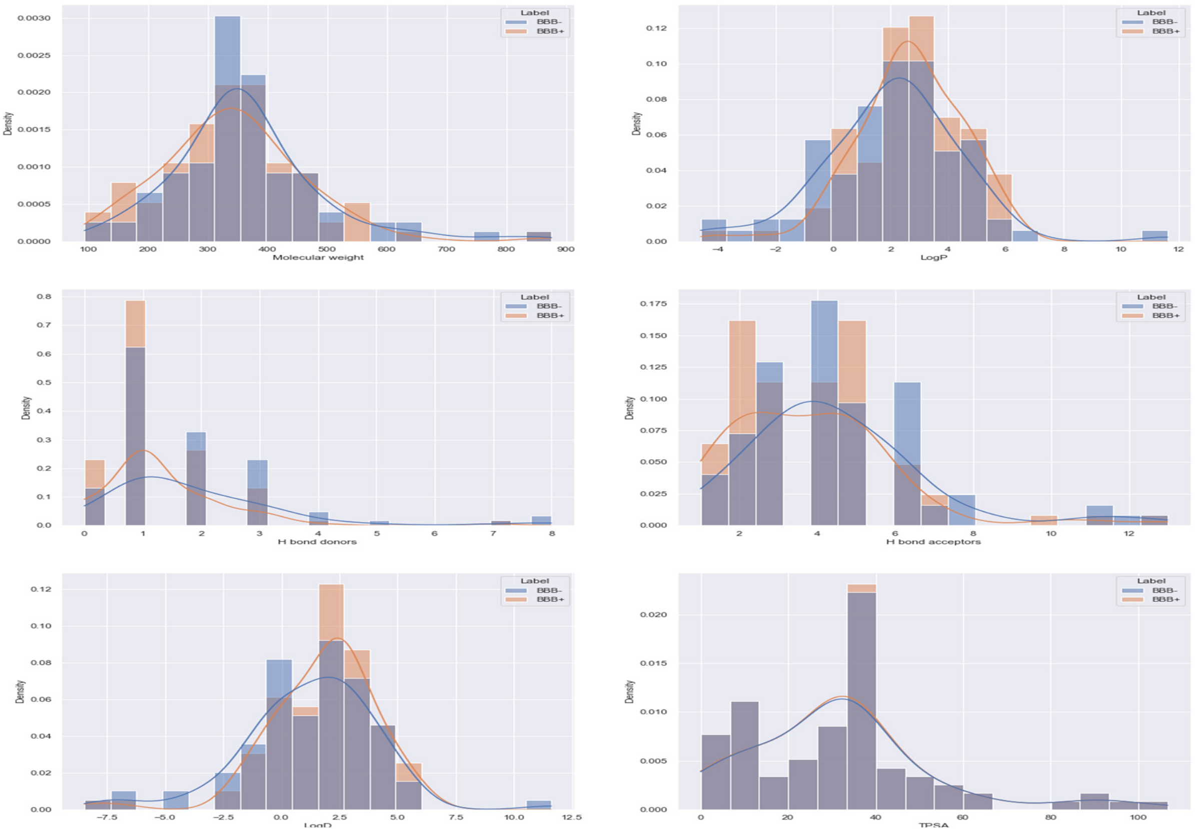

Figure S6: Trends in physiochemical properties of BBB permeable and non-permeable molecules in neutral scaffold. Density of molecules having properties in certain range is shown along y-axis and value of properties on x-axis. The Bar is colored based on permeable (orange) and non-permeable (purple). Molecular properties plotted here are i) Molecular Weight ii) LogP iii) H bond donors iv) H bond acceptors v) logD vi) TPSA.

Table S3: Models performace on MCC and AUC-ROC metrics

Models	Features	MCC Score (Train/Test)	AUC-ROC (Train/Test)
**Baseline**	DockedFP (1a)	0.013/-0.03	0.50/0.51
	DockedFP (1b)	0.006/-0.016	0.50/0.49
**SVM**	ECFP4 fingerprint	0.84/0.51	0.91/0.74
	DockedFP (1a)	0.40/0.18	0.66/0.57
	DockedFP (1b)	0.12/0.11	0.53/0.53
	Rdkit Pharmacoprint	0.75/0.48	0.85/0.72
	ECFP4+ DockedFP (1a)	0.84/0.50	0.91/0.74
	ECFP4+ DockedFP (1b)	8.84/0.50	0.91/0.74
**Random Forest**	ECFP4 fingerprint	1/0.49	1/0.73
	DockedFP (1a)	0.80/0.14	0.89/0.56
	DockedFP (1b)	0.12/0.11	0.53/0.53
	Rdkit Pharmacoprint	0.99/0.51	0.99/0.74
	ECFP4+DockedFP (1a)	1/0.47	1/0.72
	ECFP4+DockedFP (1b)	1/0.49	1/0.73
**Naïve Byes**	ECFP4 fingerprint	0.49/0.40	0.74/0.69
	DockedFP (1a)	0.12/0.11	0.54/0.54
	DockedFP (1b)	0.16/0.14	0.57/0.56
	Rdkit Pharmacoprint	0.41/0.39	0.68/067
	ECFP4+DockedFP (1a)	0.5/0.41	0.74/0.70
	ECFP4+ DockedFP (1b)	0.49/0.40	0.74/0.70
**Graph Convolution Network (GCN)**	Descriptors	0.58/0.47	0.78/0.72
**Graph Attention Network (GAT)**	Descriptors	0.67/0.51	0.83/0.75
